# Improvement of antibiotic therapy and ICU survival in severe non-pneumococcal community-acquired pneumonia: a matched case–control study

**DOI:** 10.1186/s13054-015-1051-1

**Published:** 2015-09-10

**Authors:** Simone Gattarello, Leonel Lagunes, Loreto Vidaur, Jordi Solé-Violán, Rafael Zaragoza, Jordi Vallés, Antoni Torres, Rafael Sierra, Rosa Sebastian, Jordi Rello

**Affiliations:** Critical Care Department, Vall d’Hebron Hospital, Ps. Vall d’Hebron, 119-129, Anexe AG-5a planta, 08035 Barcelona, Spain; Department of Medicine of the Universitat Autònoma de Barcelona, Vall d’Hebron Institut de Recerca (VHIR), Barcelona, Spain; Centro de Investigación Biomédica en Red de Enfermedades Respiratorias (CIBERES), Madrid, Spain; Intensive Care Department, Donostia University Hospital, Donostia, Spain; Intensive Care Department, Dr. Negrin University Hospital, Las Palmas de Gran Canaria, Spain; Intensive Care Department, Dr. Peset University Hospital, Valencia, Spain; Critical Care Centre, Sabadell Hospital, Consorci Hospitalari Universitari Parc Taulí, Sabadell, Spain; Respiratory Disease Department, Hospital Clinic i Provincial de Barcelona, University of Barcelona, Institut d’Investigacions Biomèdiques August Pi i Sunyer (IDIBAPS), Barcelona, Spain; Critical Care Unit, Puerta del Mar University Hospital, Cadiz, Spain

## Abstract

**Introduction:**

We aimed to compare intensive care unit mortality due to non-pneumococcal severe community-acquired pneumonia between the periods 2000–2002 and 2008–2014, and the impact of the improvement in antibiotic strategies on outcomes.

**Methods:**

This was a matched case–control study enrolling 144 patients with non-pneumococcal severe pneumonia: 72 patients from the 2000–2002 database (CAPUCI I group) were paired with 72 from the 2008–2014 period (CAPUCI II group), matched by the following variables: microorganism, shock at admission, invasive mechanical ventilation, immunocompromise, chronic obstructive pulmonary disease, and age over 65 years.

**Results:**

The most frequent microorganism was methicillin-susceptible *Staphylococcus aureus* (22.1 %) followed by *Legionella pneumophila* and *Haemophilus influenzae* (each 20.7 %); prevalence of shock was 59.7 %, while 73.6 % of patients needed invasive mechanical ventilation. Intensive care unit mortality was significantly lower in the CAPUCI II group (34.7 % versus 16.7 %; odds ratio (OR) 0.78, 95 % confidence interval (CI) 0.64–0.95; *p* = 0.02). Appropriate therapy according to microorganism was 91.5 % in CAPUCI I and 92.7 % in CAPUCI II, while combined therapy and early antibiotic treatment were significantly higher in CAPUCI II (76.4 versus 90.3 % and 37.5 versus 63.9 %; *p* < 0.05). In the multivariate analysis, combined antibiotic therapy (OR 0.23, 95 % CI 0.07–0.74) and early antibiotic treatment (OR 0.07, 95 % CI 0.02–0.22) were independently associated with decreased intensive care unit mortality.

**Conclusions:**

In non-pneumococcal severe community-acquired pneumonia , early antibiotic administration and use of combined antibiotic therapy were both associated with increased intensive care unit survival during the study period.

## Introduction

In Western countries, community-acquired pneumonia (CAP) is the leading cause of death and is associated with high healthcare costs [1]. In the intensive care unit (ICU) setting, it is one of the most common reasons for admission and the most frequent causes of mortality [2]. Antibiotic treatment is the cornerstone for management of pneumonia, and adequate empiric treatment is associated with improved outcomes [1].

The trend in CAP mortality over recent decades remains unclear, despite many efforts to identify it. Contrasting results have been obtained in different publications because most of them did not differentiate between outpatients, patients admitted to the ward and patients admitted to the ICU [3, 4]. Conversely, many recently published studies have found a significant decrease in mortality in septic shock [5–7], or in severe respiratory failure requiring invasive mechanical ventilation (IMV) [8]; therefore, it is reasonable to assume that mortality due to severe pneumonia, especially when complicated with septic shock, may have decreased in the last few years. In a recent study, we found a reduction in mortality due to pneumococcal severe (S)CAP and an association between better management of antibiotic therapy and improved ICU survival [9].

The present study hypothesizes that, as in pneumococcal SCAP [9], an improvement in antibiotic policies will contribute to reducing mortality in non-pneumococcal SCAP. The primary objective was to determine ICU mortality due to non-pneumococcal SCAP, and the secondary objective was to assess whether improvements in antibiotic prescription had been implemented.

## Materials and methods

This was a matched case–control study enrolling 144 ICU patients diagnosed with non-pneumococcal SCAP: 72 patients from the CAPUCI I study (2000–2002 period) were paired with 72 patients from the CAPUCI II database (2008–2014 period).

CAPUCI I was a multicenter, prospective, observational study carried out in 33 hospitals in Spain between 2000 and 2002. All patients admitted to ICU with diagnosis of SCAP were included. CAPUCI II was a follow-up project endorsed by the European Critical Care Research Network, carried out in 29 European ICUs from 2008 to 2014. In both studies, patients were admitted to the ICU either to undergo IMV or because they were in an unstable clinical condition [1]. All cases were followed until ICU discharge or death, and all clinical decisions were left to the discretion of the attending physician. Data from these cohorts have been reported elsewhere [10]. The Joan XXIII University Hospital Ethics Board (coordinating centre) approved the study (REF 2005/NA); the need for informed consent was waived due to the observational nature of the studies.

Pneumonia was diagnosed when a patient had consistent clinical findings plus a new pulmonary infiltrate on chest radiography. Immunocompromise was defined as primary immunodeficiency or immunodeficiency secondary to radiation treatment, use of cytotoxic drugs or steroids (daily doses >20 mg prednisolone or equivalent for >2 weeks), transplantation or AIDS. Shock was defined as the need for a vasopressor during >4 hours after fluid replacement; rapid radiographic spread was defined as an increase in the size of opacities on chest radiograph >50 % at 48 hours.

SCAP was defined as pneumonia that required ICU admission, with single or multi-organ failure. Patients proceeding from a long-term care facility, diagnosed with healthcare-associated pneumonia and with a no-cardiopulmonary resuscitation indication were not included. Microbiological diagnosis required a positive result from a respiratory sample or blood culture, or a positive urinary antigen in the case of *Legionella* spp*.* infection. Probability of death was predicted according to the “estimated risk of mortality” using the Acute Physiology and Chronic Health Evaluation (APACHE) II score in the CAPUCI I cohort and Simplified Acute Physiology Score (SAPS) III in the CAPUCI II cohort [11, 12]. Monotherapy and combined therapy were defined as administration of the same antibiotic (one or more) during the first 2 days of ICU admission. Early antibiotic administration was defined as administration of the first dose of antibiotic within 3 hours of hospital admission.

Patients with pneumonia and negative cultures, documented viral pneumonia or mixed aerobic/anaerobic flora were excluded; likewise, aspiration pneumonia, often associated with impaired clinical status [13], was excluded from the analysis so as to avoid bias. To perform the case–control analysis, each patient from the CAPUCI II group with a confirmed microbial etiology was matched with one from the CAPUCI I group with the same microorganism. Subsequently, the rest of variables used to match patients were: 1) presence of shock at ICU admission; 2) need for IMV; 3) immunosuppression; 4) chronic obstructive pulmonary disease; and 5) age (cut-off, 65 years) [14]; all main determinants for mortality in CAP [15, 16].

All data management and statistical analyses were performed using the SPSS 20 processor (SPSS inc., Chicago, IL). Results are expressed as medians and interquartile range for continuous variables, or as absolute percentages for categorical variables. Continuous variables were compared with the Mann–Whitney U test (non-normally distributed variables). Categorical variables were assessed with the chi-square or two-tailed Fisher exact test.

A multivariate model was performed to identify the variables associates with changes in mortality. To construct the model, we performed a logistic regression using all variables from the univariate analysis that were associated with a different mortality as covariates; subsequently, to optimize the model and minimize an overfitting bias, an automatic stepwise backward covariate selection was performed. Thus, multivariate analysis was finally adjusted according to the following variables: shock at admission, acute renal failure, combination therapy and early antibiotic administration.

Kaplan-Meier analysis was used to construct survival curves for patients receiving combination and monotherapy regimens and early versus late antibiotic administration.

## Results

**Fig. 1:**
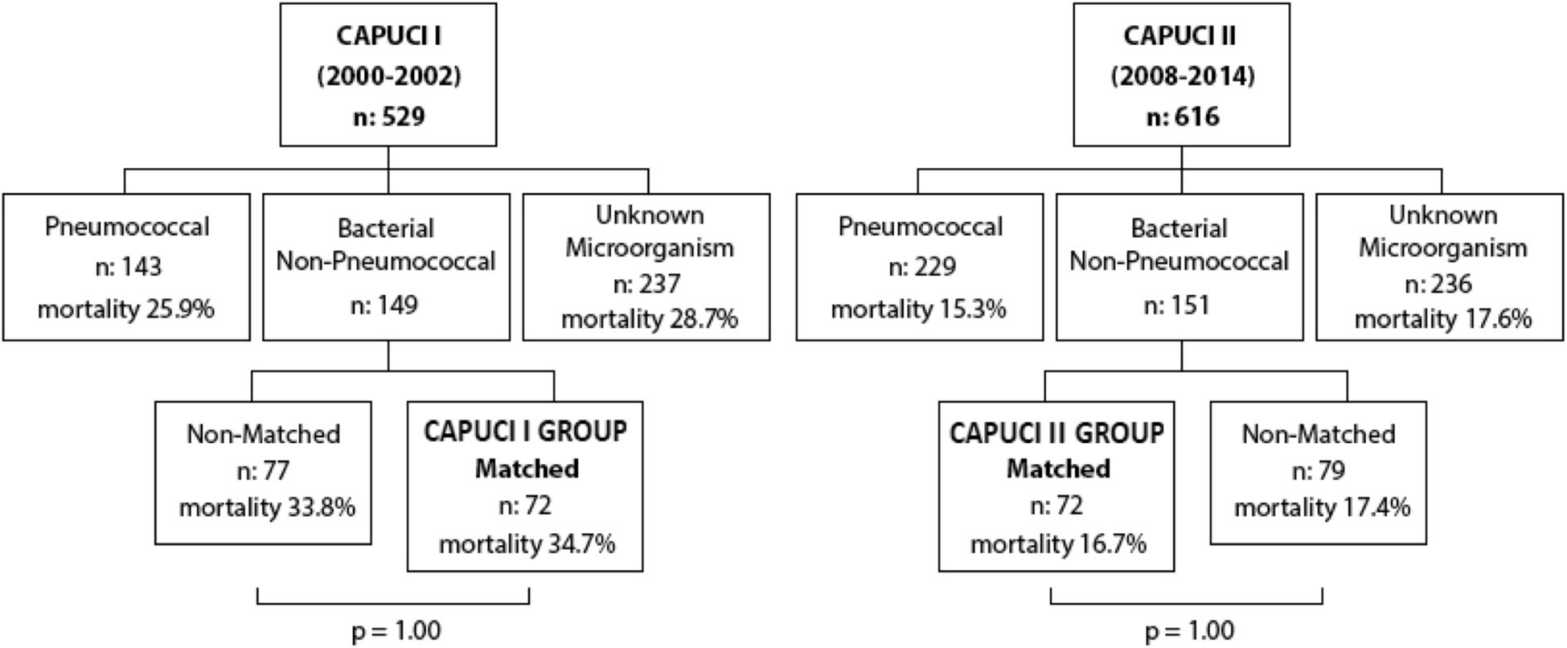
Flow chart diagram of patient selection and mortality in the different subgroups

**Fig. 2:**
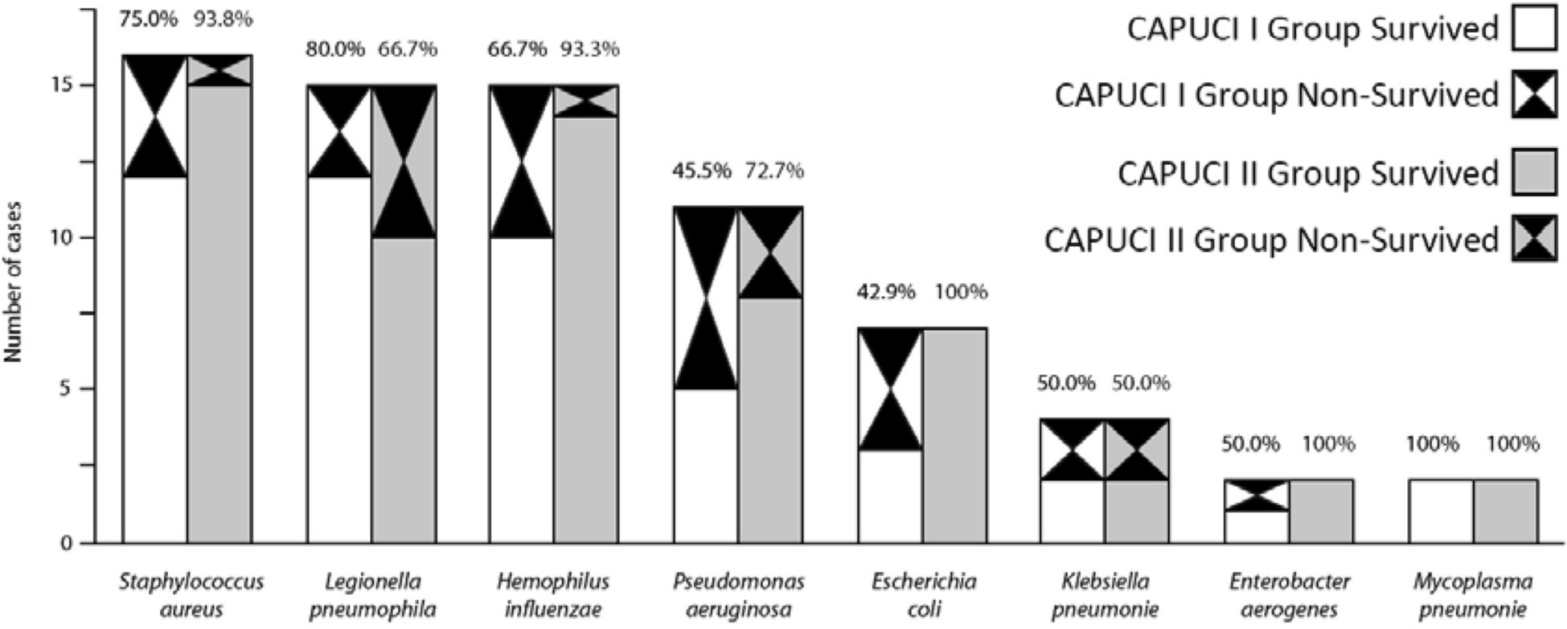
ICU survival according to microorganism

**Fig. 3:**
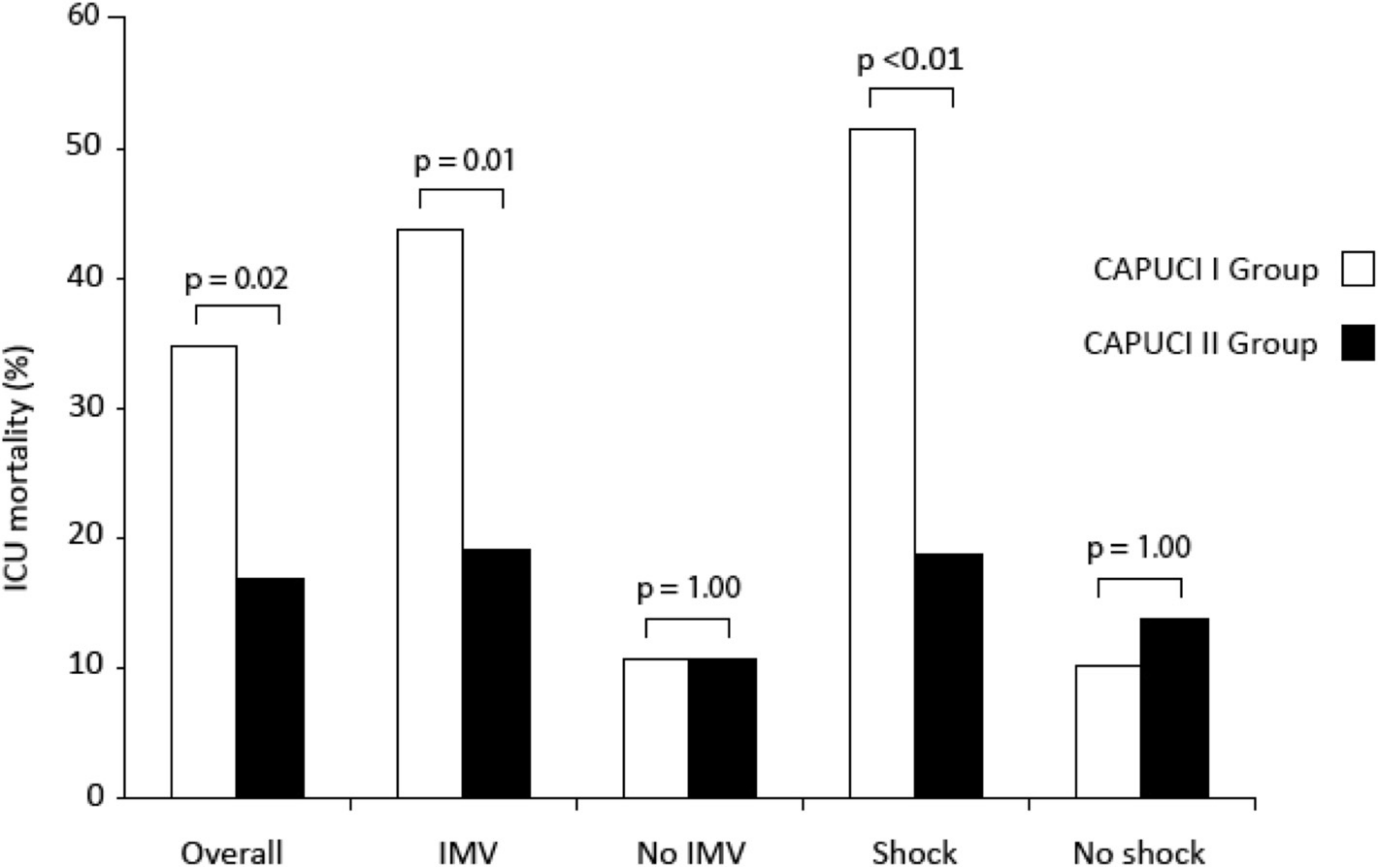
ICU mortality in the whole population and in different subgroups of patients. *ICU* intensive care unit, *IMV* invasive mechanical ventilation

ICU mortality was significantly lower in the CAPUCI II group (34.7 versus 16.7 %; odds ratio (OR) 0.78, 95 % confidence interval (CI) 0.64–0.95; *p* = 0.02). Figure 1 shows a flow chart analysis for patient selection; in both CAPUCI I and CAPUCI II populations, mortality among patients enrolled in the analysis was compared with mortality of the rest of patients in the same group, with no significant differences being found (Fig. 1). In both cohorts, mortality of patients enrolled in the case–control analysis did not present significant differences when compared with patients not introduced into the analysis. Mortality in the CAPUCI I cohort was 34.7 % in patients enrolled in the analysis versus 33.8 % in non-matched patients with non-pneumococcal pneumonia with confirmed microorganism (*p* = 1.00); in CAPUCI II, it was 16.7 % in matched and 17.4 % in non-matched patients, respectively (*p* = 1.00). Figure 2 depicts survival depending on the microbiological isolate: the most frequent microorganism was *Staphylococcus aureus* (22.1 %), followed by *Legionella pneumophila* and *Haemophilus influenzae* (each 20.7 %). Table 1 shows the variables used to pair patients; individuals selected for the case–control analysis showed a prevalence of shock and IMV of 59.7 % and 73.6 %, respectively. Furthermore, Table 1 shows the prevalence of the matching variables in patients with non-pneumococcal bacterial pneumonia not selected for the matching analysis; no significant differences were observed between matched and non-matched patients. As indicated in Table 2, estimated probability of death was 32.0 % in the CAPUCI II group and 34.0 % in the CAPUCI I group (*p* = 0.59). Bacteremia was observed in 22.2 % of CAPUCI I and in 30.6 % of CAPUCI II (*p* = 0.35). No significant differences were observed in length of ICU stay (10.5 versus 12.0 days; *p* = 0.16) or length of IMV (8.0 versus 10.5 days; *p* = 0.18). Mortality showed an absolute reduction of 18 % between the CAPUCI II group (34.7 %) and the CAPUCI I group (16.7 %) (*p* = 0.02). Figure 3 compares ICU mortality between groups. In the CAPUCI II group, it fell significantly in the overall population with an OR of 0.78 (95 % CI 0.64–0.95; *p* = 0.02); in ventilated patients the OR was 0.70 (95 % CI 0.53–0.91; *p* = 0.01) while in patients with shock it was 0.60 (95 % CI 0.43–0.84; *p* < 0.01).

**Table 1:** Description of matched variables and mortality

Variable	CAPUCI I group	CAPUCI I	*p* value	CAPUCI II group	CAPUCI II	*p* value
	(*n* = 72)	non-matched		(*n* = 72)	non-matched	
Age over 65 years	32 (44.4)	22 (28.6)	0.06	32 (44.4)	23 (41.8)	0.86
COPD	34 (47.2)	24 (31.2)	0.06	34 (47.2)	19 (36.5)	0.27
Immunosuppression	9 (12.5)	17 (22.1)	0.14	9 (12.5)	7 (12.5)	1.00
Shock	43 (59.7)	47 (61.0)	1.00	43 (59.7)	24 (43.6)	0.08
IMV	53 (73.6)	57 (74.0)	1.00	53 (73.6)	32 (60.4)	0.13
ICU mortality	25 (34.7)	26 (33.8)	1.00	12 (16.7)	8 (17.4)	1.00

Data are presented as n (%). *COPD* chronic obstructive pulmonary disease, *ICU* intensive care unit, *IMV* invasive mechanical ventilation

**Table 2:** Other demographical data and clinical presentations

Variable	CAPUCI I group	CAPUCI II group	*p* value
	(*n* = 72)	(*n* = 72)	
Age^a^	63.0 (47.5–75.0)	62.0 (53.0–72.0)	0.85
Age under 50 years	20 (27.8)	14 (19.4)	0.33
Age 50–64 years	20 (27.8)	24 (33.3)	0.59
Age 65–74 years	13 (18.1)	20 (27.8)	0.23
Age over 75 years	19 (26.4)	14 (19.4)	0.43
Male gender	49 (68.1)	54 (75.0)	0.46
Active smoker	25 (34.7)	27 (37.5)	0.86
Alcohol use	24 (33.3)	14 (19.4)	0.09
Overweight	14 (19.4)	21 (29.2)	0.24
Diabetes mellitus	18 (25.0)	15 (20.8)	0.69
Cardiomyopathy	24 (33.3)	17 (23.6)	0.27
Cerebral vascular disease	4 (5.6)	7 (9.7)	0.53
Malignancy	6 (8.3)	4 (5.6)	0.75
Estimated probability of death^a^	32.0 (19.5–50.0)	34.0 (16.0–62.8)	0.59
ICU length of stay^a^	10.5 (6.0–20.8)	12.0 (7.0–33.0)	0.16
Days of mechanical ventilation^a^	8.0 (4.0–15.3)	10.5 (6.0–23.3)	0.18
Bacteremia	16 (22.2)	22 (30.6)	0.35
Acute kidney injury	31 (43.1)	29 (40.3)	0.87
Rapid radiographic spread	40 (55.6)	37 (51.4)	0.74
ICU mortality	25 (34.7)	12 (16.7)	**0.02**

Data are presented as n (%), unless otherwise indicated: ^a^ median (interquartile range 25–75). *ICU* intensive care unit. Significant *p* values are indicated in bold

**Fig. 4:**
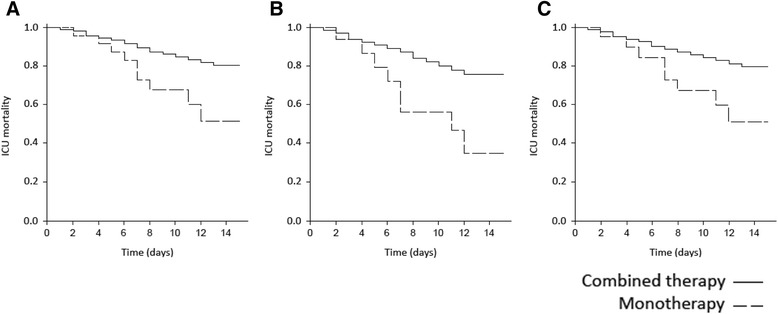
Kaplan-Meier survival curve stratified for monotherapy versus combined therapy. **a** The whole population (log rank *p* < 0.01); **b** patients with shock (log rank *p* < 0.01); **c** patients under mechanical ventilation (log rank *p* < 0.01). *ICU* intensive care unit

**Fig. 5:**
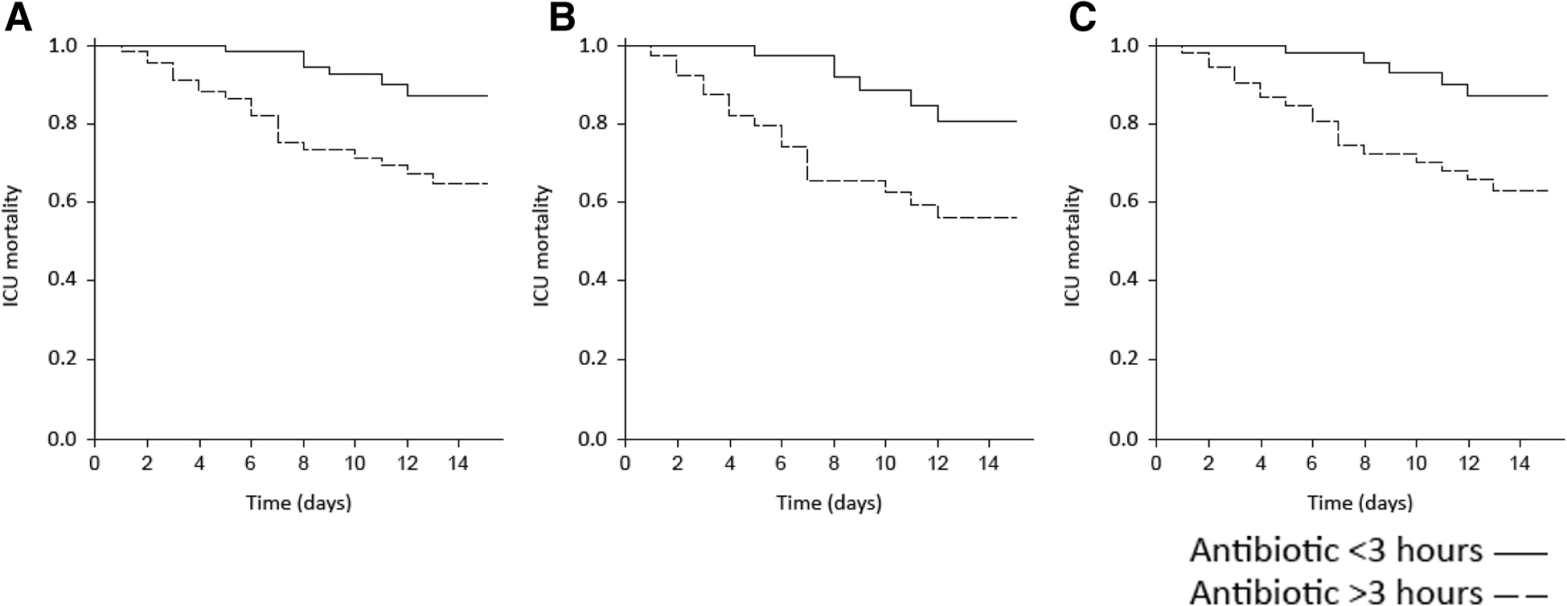
Kaplan-Meier survival curve stratified for early versus non-early antibiotic treatment. **a** The whole population (log rank *p* < 0.01); **b** patients with shock (log rank *p* < 0.01); **c** patients under mechanical ventilation (log rank *p* < 0.01). *ICU* intensive care unit

Kaplan-Meier survival analysis was performed in the whole population and in the subgroups of patients who underwent IMV or required vasopressors, stratifying by monotherapy versus combined therapy (Fig. 4; log rank *p* value <0.01 in the three analyses) and early versus non-early antibiotic treatment (Fig. 5; log rank *p* value <0.01 in all three cases). As shown in Table 3, no significant ldifferences were observed between bacteremic and non-bacteremic patients.

**Table 3:** Comparison between bacteremic and non-bacteremic patients

Variable	Bacteremic	Non-bacteremic	*p* value
	(*n* = 38)	(*n* = 106)	
Age under 50 years	9 (23.7)	25 (23.6)	1.00
Age 50–64 years	13 (34.2)	31 (29.2)	0.68
Age 65–74 years	6 (15.8)	27 (25.5)	0.27
Age over 75 years	10 (26.3)	23 (21.7)	0.65
Immunocompromised	7 (18.4)	11 (10.4)	0.25
Shock at ICU admission	23 (60.5)	63 (59.4)	1.00
Invasive mechanical ventilation	28 (73.7)	78 (73.6)	1.00
Acute kidney injury	17 (44.7)	43 (40.6)	0.70
Rapid radiographic spread	24 (63.2)	53 (50.0)	0.19
Combined therapy	34 (89.5)	86 (81.1)	0.31
Antibiotic initiated 0 to 3 hours	18 (47.4)	55 (51.9)	0.71
ICU mortality	11 (28.9)	26 (24.5)	0.67

Data are presented as n (%). *ICU* intensive care unit

Combined therapy was administered in 76.4 % of patients in the CAPUCI I group and in 90.3 % in the CAPUCI II group (*p* = 0.04) (Table 4). Early antibiotic treatment was also significantly higher in the CAPUCI II group (63.9 versus 37.5 %; *p* < 0.01). The most frequent antibiotic prescription was a cephalosporin plus a macrolide (Table 5) in 58 out of 144 patients (40.1 %), with ceftriaxone/cefotaxime plus clarithromycin being the most frequent combination (29 patients out of 144, 20.1 %). The most frequent pattern delivered in the CAPUCI I group was ceftriaxone/cefotaxime plus clarithromycin (27 patients out of 72, 37.5 %) while in the CAPUCI II group it was ceftriaxone/cefotaxime plus levofloxacin (21 patients out of 72, 17.3 %). Azithromycin was not available in Spain in parenteral formulation between 2000 and 2002, so it was not used in this group.

**Table 4:** Characteristics of antibiotic treatment

Variable	CAPUCI I group	CAPUCI II group	*p* value
	(*n* = 72)	(*n* = 72)	
Previous antibiotic	14 (19.4)	10 (13.9)	0.50
Monotherapy	17 (23.6)	7 (9.7)	**0.04**
Combined therapy	55 (76.4)	65 (90.3)	**0.04**
Antibiotic initiated 0 to 3 hours	25 (37.5)	46 (63.9)	**<0.01**
Antibiotic initiated 4 to 6 hours	21 (29.2)	17 (23.6)	0.57
Antibiotic initiated more than 6 hours	25 (33.3)	9 (12.5)	**<0.01**
Adequate according to 2007 IDSA/ATS guidelines	31 (43.1)	41 (56.9)	0.13

Data are presented as n (%). Significant *p* values are indicated in bold. *IDSA/ATS* Infectious Diseases Society of America/American Thoracic Society

**Table 5:** Most frequent patterns of antibiotic treatment

Variable	All patients	CAPUCI I group	CAPUCI II group	*p* value
	(*n* = 144)	(*n* = 72)	(*n* = 72)	
Cephalosporin and macrolide	58 (40.1)	35 (48.5)	23 (32.0)	0.06
Ceftriaxone/cefotaxime and clarithromycin	29 (20.1)	27 (37.5)	2 (2.8)	**<0.01**
Ceftriaxone/cefotaxime and azithromycin	18 (12.4)	0 (0)	18 (25.0)	**<0.01**
Other cephalosporin and macrolide	11 (7.6)	8 (11.1)	3 (4.2)	0.21
Cephalosporin and quinolone	31 (21.5)	6 (8.4)	25 (34.6)	**<0.01**
Cefotaxime/ceftriaxone and levofloxacin	25 (17.3)	4 (5.6)	21 (29.0)	**<0.01**
Other cephalosporin and quinolone	6 (4.2)	2 (2.8)	4 (5.6)	0.68
Penicillin and macrolide	9 (6.3)	2 (2.8)	7 (9.7)	0.17
Piperacillin-tazobactam and azithromycin	5 (3.5)	0 (0)	5 (6.9)	0.06
Other penicillin and macrolide	4 (2.8)	2 (2.8)	2 (2.8)	1.00
Amoxicillin-clavulanate	8 (5.6)	8 (11.1)	0 (0)	**<0.01**
Ceftriaxone/cefotaxime	7 (4.9)	5 (6.9)	2 (2.8)	0.44
Levofloxacin	6 (4.2)	4 (5.6)	2 (2.8)	0.68
Miscellaneous combined therapy	22 (15.3)	12 (16.7)	10 (13.9)	0.78
Miscellaneous monotherapy	3 (2.1)	0 (0)	3 (4.2)	0.25
Overall	144 (100)	72 (100)	72 (100)	

Data are presented as n (%). Significant *p* values are indicated in bold. *p* value calculated between CAPUCI I and CAPUCI II groups

Table 6 shows the empiric antibiotic treatment delivered to each patient, and the appropriateness of the empiric antibiotic treatment with respect to the isolated microorganism. Overall, appropriate therapy based on bacteriology was 91.5 % in CAPUCI I and 92.7 % in CAPUCI II. Appropriate therapy was prescribed in all episodes caused by *Staphylococcus aureus*, *Haemophilus influenzae*, *Escherichia coli*, *Klebsiella pneumoniae*, *Mycoplasma pneumoniae* and *Enterobacter aerogenes*. Inappropriate therapy was only prescribed in episodes caused by *Legionella pneumophila* (2/15 and 2/15 in CAPUCI I and II, respectively) and *Pseudomonas aeruginosa* (6/11 and 5/11 in CAPUCI I and II, respectively).

**Table 6:** Treatment of each case of pneumonia and rate of adequate treatment according with the isolated microorganism

	*Staphylococcus aureus*		*Legionella pneumophila*		*Haemophilus influenzae*		*Pseudomonas aeruginosa*		*Escherichia coli*		*Klebsiella pneumoniae*		*Mycoplasma pneumoniae*		*Enterobacter aerogenes*	
	C I	C II	C I	C II	C I	C II	C I	C II	C I	C II	C I	C II	C I	C II	C I	C II
Amoxicillin-clavulanate	1 (0)	–	1 (1)	–	5 (3)	–	1 (1)	–	–	–	–	–	–	–	–	–
Cefotaxime/ceftriaxone	3 (1)	–	1 (0)	2 (2)	–	–	–	–	–	–	–	–	–	–	1 (1)	–
Levofloxacin	1 (0)	–	–	–	–	1 (0)	1 (1)	1 (1)	2 (1)	–	–	–	–	–	–	–
Piperacillin-tazobactam	–	1 (0)	–	–	–	–	–	1 (1)	–	1 (0)	–	–	–	–	–	–
Amoxicillin-clavulanate plus macrolide	–	–	1 (0)	1 (1)	–	–	–	–	–	–	–	–	–	–	–	–
Amoxicillin-clavulanate plus clindamycin	–	–	–	–	–	–	–	–	–	1 (0)	–	–	–	–	–	1 (0)
Amoxicillin-clavulanate plus levofloxacin	–	1 (0)	–	–	–	–	–	–	–	–	–	1 (1)	–	–	–	–
Cefotaxime/ceftriaxone plus macrolide	9 (2)	7 (0)	6 (0)	5 (2)	5 (2)	3 (0)	4 (2)	2 (0)	1 (1)	2 (0)	3 (1)	–	1 (0)	1 (0)	–	–
Cefotaxime/ceftriaxone plus aztreonam	–	–	–	–	–	–	1 (0)	–	–	–	–	–	–	–	–	–
Cefotaxime/ceftriaxone plus clindamycin	–	–	–	–	–	–	–	–	–	–	–	–	–	–	–	–
Cefotaxime/ceftriaxone plus levofloxacin	1 (1)	2 (0)	1 (1)	5 (0)	–	6 (0)	–	2 (0)	1 (0)	3 (0)	1 (1)	1 (0)	–	1 (0)	–	1 (0)
Cefepime/ceftazidime plus macrolide	–	1 (0)	1 (0)	1 (0)	3 (0)	1 (0)	1 (1)	–	1 (1)	–	–	–	–	–	–	–
Cefepime plus amikacin	–	–	–	–	–	–	–	–	1 (1)	–	–	–	–	–	–	–
Cefepime plus ciprofloxacin	–	–	1 (0)	–	1 (0)	–	–	–	–	–	–	–	–	–	–	–
Cefepime plus levofloxacin	–	2 (1)	–	–	–	2 (0)	–	–	–	–	–	–	–	–	–	–
Carbapenem/piperacillin-tazobactam plus amikacin	–	–	–	–	–	–	1 (0)	–	–	–	–	–	–	–	–	–
Carbapenem/piperacillin-tazobactam plus clindamycin	–	1 (0)	–	–	1 (0)	–	–	–	–	–	–	–	–	–	–	–
Carbapenem/piperacillin-tazobactam plus ciprofloxacin	1 (0)	–	–	–	–	–	2 (1)	–	–	–	–	–	1 (0)	–	–	–
Carbapenem/piperacillin-tazobactam plus macrolide	–	1 (0)	1 (1)	–	–	1 (1)	–	4 (1)	–	–	–	–	–	–	1 (0)	–
Levofloxacin plus azithromycin	–	–	–	1 (0)	–	1 (0)	–	–	–	–	–	–	–	–	–	–
Levofloxacin plus tobramycin	–	–	–	–	–	–	–	–	1 (0)	–	–	–	–	–	–	–
Piperacillin-tazobactam plus levofloxacin	–	–	2 (0)	–	–	–	–	1 (0)	–	–	–	2 (1)	–	–	–	–
Overall	16	16	15	15	15	15	11	11	7	7	4	4	2	2	2	2
Adequate according with microorganism^a^	16 (100)	16 (100)	13 (86.7)	13 (86.7)	15 (100)	15 (100)	5 (45.5)	6 (54.5)	7 (100)	7 (100)	4 (100)	4 (100)	2 (100)	2 (100)	2 (100)	2 (100)

Data are shown as absolute count of patients that received a specific antibiotic regimen; the number of patients who died receiving this antibiotic regimen is shown between parenthesis. ^a^ Data are presented as *n* (%). *C I* CAPUCI I, *C II* CAPUCI II

Table 7 shows univariate and multivariate analyses for the assessment of variables associated with different mortality rates. In the univariate analysis, variables associated with a significant rise in ICU mortality were shock at ICU admission (*p* < 0.01), acute kidney injury (*p* < 0.01), need for IMV (*p* = 0.02) and alcohol use (*p* = 0.03); factors associated with lower ICU mortality were combined therapy (*p* < 0.01) and early antibiotic treatment (*p* < 0.01).

**Table 7:** Univariate and multivariate analyses to assess variables associated with changes in ICU mortality

Variable	Survival	No survival	Univariate analysis	Multivariate analysis:
	(*n* = 107)	(*n* = 37)	*p* value	OR (95 % CI); *p* value
Age over 65 years	46 (43.0)	18 (48.6)	0.57	
Overweight	27 (25.2)	8 (21.6)	0.83	
Alcohol use	23 (21.5)	15 (40.5)	**0.03**	
Active smoker	36 (33.6)	16 (43.2)	0.33	
Diabetes mellitus	24 (22.4)	9 (24.3)	0.83	
Cardiomyopathy	28 (26.2)	13 (35.1)	0.30	
COPD	49 (45.8)	19 (51.4)	0.57	
Immunosuppression	13 (12.1)	5 (13.5)	0.78	
Shock at ICU admission	56 (52.3)	30 (81.1)	**<0.01**	3.96 (1.29–12.14); **0.02**
Invasive mechanical ventilation	73 (68.2)	33 (89.2)	**0.02**	
Acute kidney injury	36 (33.6)	24 (64.9)	**<0.01**	4.56 (1.60–13.02); **<0.01**
Rapid radiographic spread	56 (52.3)	21 (56.8)	0.70	
Bacteremia	27 (25.2)	11 (29.7)	0.67	
Combined therapy	96 (89.7)	24 (64.9)	**<0.01**	0.23 (0.07–0.74); **0.01**
AB initiated within 3 hours	67 (62.6)	6 (16.2)	**<0.01**	0.07 (0.02–0.22); **<0.01**
Combined BL and M therapy	52 (48.6)	15 (40.5)	0.45	
Combined BL and FQ therapy	33 (30.8)	6 (16.2)	0.09	

Data are presented as n (%). Significant *p* values are indicated in bold. *AB* antibiotic, *BL* beta-lactam, *CI* confidence interval, *COPD* chronic obstructive pulmonary disease, *FQ* fluoroquinolone, *ICU* intensive care unit, *M* macrolide, *OR* odds ratio

As shown in Table 7, we explored whether the administration of a specific antibiotic combination was associated with changes in mortality, without observing significant differences. We compared mortality after the administration of either beta-lactam-macrolide (univariate analysis: OR 0.72, 95 % CI 0.34–1.54) or beta-lactam-quinolone (univariate analysis: OR 0.43, 95 % CI 0.17–1.14) regimens, without observing significant differences (data not shown).

Variables from the univariate analysis that were associated with significant changes in mortality were introduced in a multivariate model analysis. Acute kidney injury and shock at ICU admission were associated with a higher risk of ICU mortality (OR 4.56, 95 % CI 1.60–13.02; and OR 3.96, 95 % CI 1.29–12.14, respectively. Conversely, early antibiotic treatment (OR 0.07, 95 % CI 0.02–0.22) and combined therapy (OR 0.23, 95 % CI 0.07–0.74) were associated with a lower risk of mortality during ICU admission.

Furthermore, we explored if the agreement with 2007 Infectious Diseases Society of America/American Thoracic Society (IDSA/ATS) guidelines was associated with an improved outcome, observing a decreased mortality after delivery of adequate treatment (OR 0.65, 95 % CI 0.48–0.89). When we performed a multivariate analysis (variable introduced in the model: shock at admission, acute renal failure and 2007 IDSA/ATS agreement) the association was still significantly present (OR 0.39, 95 % CI 0.17–0.91). However, the same association was not present when, in the same multivariate model, the variables “combination therapy” and “early antibiotic administration” were added (for mortality for the variable “2007 IDSA/ATS agreement: OR 0.98; 95 % CI 0.32–2.97).

## Discussion

The most relevant conclusions of the present analysis were the significant decrease in ICU mortality due to non-pneumococcal SCAP between the two cohorts, and the positive association between improved empirical antibiotic treatment and a lower mortality rate. These findings confirm our primary hypothesis, and are consistent with the results obtained from our previous study in patients with pneumococcal CAP from the same database [9].

Few published studies have assessed changes in mortality due to CAP in recent years. Moreover, as mentioned above, the vast majority did not differentiate between critical and non-critical patients [3, 4]. However, several studies have reported a significant decrease in mortality due to all-source septic shock in recent decades [5–7]. Our results were obtained in a population with a high rate of IMV (73.6 %) or secondary shock (59.7 %). In this setting, during the planning of a study, we believe that is vital to differentiate between critical and non-critical patients.

To our knowledge, no studies to date have assessed mortality due to SCAP with regard to etiology. It is worth noting that, during the matching process, we did not set a percentage for each microorganism a priori; instead, we found the number of coincidences between groups according to the above-mentioned variables. Interestingly, the distribution of the identified microorganisms in our sample coincides with literature reports, thus confirming the study’s external reproducibility [1, 17].

As indicated in Table 6, the vast majority of patients received adequate treatment according to microbiology. As expected, appropriateness of treatment in case of infection due to *Legionella pneumophila* and *Pseudomonas aeruginosa* was lower. In case of *Legionella* infection the treatment was adequate in 86.7 % of patients from the CAPUCI I and II groups. On the other hand, treatment was adequate with respect to microbiology respectively in 45.5 and 54.5 % of patients with infection due to *Pseudomonas aeruginosa*. This is important because an inappropriate treatment can lead to an increased mortality. In fact, the survival rate in case of *Pseudomonas* pneumonia was the lowest when compared with other etiologies (with the exception of *Enterobacter* infection; however, this is not significant as only four patients were introduced in the analysis, two in each group), and this is probably due to the low rate of adequate empirical treatment. Despite this, an improvement in mortality was observed between the two periods, and this is probably due to the earlier administration of the first dose of antibiotic and other general improvements that have been made in the last years in the management of ICU patients.

When exploring mortality depending on the etiology of pneumonia, we observed an increased survival between the two cohorts when pneumonia was caused by *Staphylococcus aureus*, *Haemophilus influenzae*, *Pseudomonas aeruginosa, Escherichia coli* and *Enterobacter aerogenes*. No changes were observed in case of *Klebsiella* infection, and an increased mortality was observed in case of *Legionella* pneumonia. As shown in Table 3, a higher proportion of patients from CAPUCI II received early combination therapy. When exploring the administration of mono- versus combination therapy, differentiating by the microorganisms, it becomes evident that all cases with the exception of *Legionella* and *Pseudomonas* infections received a higher rate of combined therapy if belonging to the CAPUCI II group (Table 6). Thus, it is reasonable to assume that the decrease in mortality in our sample was mainly caused by a higher rate of combination therapy and early antibiotic administration.

In the case of pneumonia due to *Legionella pneumophila*, an increased mortality between the two groups was observed. Fifteen patients with *Legionella* infection were included in both groups: 3 out of 15 and 5 out 15 died, respectively, in the CAPUCI I and II groups. Among those who died, two individuals out of three from the CAPUCI I cohort received microbiological adequate coverture (one received a quinolone regimen while the other a macrolide regimen); in CAPUCI II, they were three out of five (all received a macrolide regimen) (Table 6). On the other hand, all patients but one (belonging to the CAPUCI II group) received delayed antibiotic treatment. We assume that the small size of the sample, fortuity and probably some unrecognized factor other than antibiotic therapy played a role in the different mortality. Due to the low number of patients that died because of *Legionella* infection, it is difficult to ascribe changes in mortality because of the use of quinolones rather than macrolides, although it is not possible to exclude it.

In the case of *Klebsiella* infection, due to the reduced number of cases (four patients for each group, with 50 % mortality in both cohorts), it is not possible to take definitive conclusions.

Another important finding is the association between lower mortality and an improvement in antibiotic strategies; that is, combined antibiotic therapy and early treatment initiation. Combined therapy has become common practice in the empirical treatment of SCAP [1]. To our knowledge, no current guideline suggests monotherapy as empirical therapy. Generally, the recommendation is a beta-lactam plus either a macrolide or a quinolone. In the CAPUCI I group, monotherapy was administered in 17 patients (23.6 %) and in 7 patients (9.7 %) in the CAPUCI II group.

Univariate and multivariate analysis, and Kaplan-Meier survival analysis, all showed a positive association between delivery of combined antibiotic therapy and lower mortality. Previous studies observed that patients with CAP and secondary shock or bacteremia [18, 19] presented lower mortality when combined therapy was delivered. Our results confirm these findings and, furthermore, suggest that all patients with severe pneumonia, with or without shock or need for IMV, may benefit from combined therapy. Even though this may not be a surprising conclusion, it should be noted that a significant percentage of our population and those in other studies still received monotherapy [20]. These findings are consistent with those reported in patients with pneumococcal SCAP [9].

To date, the optimal antibiotic combination choice in severe CAP is still a debated issue; some authors advocate the use of a macrolide-regimen administration, due to the anti-inflammatory effects shown by these molecules [21]. However, not all studies achieved similar results. Although in our cohort the limited size of the sample makes it difficult to explore this issue, we assessed changes in mortality after macrolide or quinolone administration, in the whole population (Table 7) and in the subgroups of patients with shock or under mechanical ventilation (data not shown)—no differences were observed. Furthermore, several studies concluded that quinolone administration is comparable in terms of mortality with a macrolide regimen, but with a higher eradication rate, a lower treatment failure and possibly less cost of treatment [22]; however, concerns about an increased resistance rate after quinolone administration were raised [23]. Moreover, in case of a social environment with high rates of pulmonary tuberculosis, the use of a fluoroquinolone could mask a pulmonary tuberculosis rather than bacterial CAP [24]. In our sample, the use of a quinolone regimen showed a trend to lower mortality, without achieving significant results, in the whole population and in the subgroups of patients with shock or under mechanical ventilation. According to the present results, it is not possible to advocate the use of a specific antibiotic family.

In 2006, Kumar et al. showed that mortality rates in septic patients with shock increased in line with the delay in antibiotic initiation [25]. Subsequent other studies of patients with shock confirmed this finding [26]. As shown in the Kaplan-Meier survival analysis (Fig. 5), early antibiotic treatment in our sample was associated with lower mortality not only in the subgroup of patients with shock, but in the overall population and in the subgroup of ventilated patients as well. In view of these findings, each patient who presents at the emergency room with non-pneumococcal SCAP should receive the first dose of antibiotic treatment within the first 3 hours. Indeed, the 2007 IDSA/ATS guidelines recommend initiation of antibiotic therapy before transfer to the ward or to the ICU [1].

We explored if the agreement with the 2007 IDSA/ATS guidelines was associated with different outcomes, and we found a reduced mortality when guideline recommendations were followed. Basically, IDSA/ATS guidelines recommend the administration of an early combination of specific antibiotic families; thus, to better investigate the effects of simple medical actions on mortality, we decided to separate the item “2007 IDSA/ATS adequate treatment” into two variables: “combined therapy” and “early antibiotic treatment”. Thus, after the addition of these two variables in the multivariate model this association was no longer documented. This might suggest that the sum of actions that imply the agreement to international guidelines is better investigated by separating these actions in multiple variables rather that grouping all actions into the variable “2007 IDSA/ATS adequate treatment”.

The current study has some limitations that should be mentioned. The most important is its observational nature; however, this approach is the only way to assess changes in outcome over a period of time. Moreover, in the ICU setting in recent decades there have been significant improvements in the management of patients undergoing IMV or resuscitation of septic shock, and in nutrition, in the prevention of ICU-related complications and in the ICU admission criteria as well. Severity-of-illness was recorded with different scores; in CAPUCI I the risk of mortality was estimated using APACHE II score while in CAPUCI II SAPS III was used. However, both scales reflect a reliable risk of death and were widely validated in large-scale studies. For this, after estimating the risk of death with APACHE II and SAPS III scores, we created a variable named “estimated probability of death”, comparing both groups. Furthermore, to avoid an overlap between the same parameters in different variables, we decided not to use severity-score variables to match patients; in fact, both APACHE II and SAPS III scores include parameters of severity for respiratory or cardiac failure, and demographics such as age, which were introduced independently in the matching. Of note, the ICUs that participated in CAPUCI I and II studies were different; in fact, CAPUCI I was developed in 33 Spanish ICUs while CAPUCI II was carried out in 29 ICUs–24 from Spain and 5 from other European countries. This may originate a bias due to a different source of patients; however, after the matching process, we retrospectively observed that all 72 patients from the CAPUCI II cohort were enrolled from Spanish ICUs, thus minimizing the risk of a significant bias. Moreover, we did not perform genetic investigations, although recent publications have identified common variants in specific genes that are associated with different outcomes in severe pneumonia [27]. Finally, as stated by Waterer [28], ICU outcome often differs from hospital outcome or from outcome on day 60 or 90 after admission. However, as shown in the Kaplan-Meier survival analysis, the majority of deaths in our sample occurred within 14 days of admission. Although it is true that ICU mortality may differ from hospital or 3-month outcome, we feel that deferring excessively the time of the study of the outcome may complicate the analysis, because more variables that are difficult to record and interpret have to be introduced in the analysis as confounding variables.

## Conclusions

In the current study, mortality due to non-pneumococcal SCAP decreased between the two cohorts, and the use of combined antibiotic therapy and early antibiotic administration were associated with lower mortality. These finding agree with the conclusions obtained in a previous study carried out in patients with pneumococcal SCAP from the same database [9]. More studies to confirm these findings are now needed. In the meantime, all patients presenting at the emergency department with SCAP should receive two antibiotics within 3 hours of admission.

## Key messages

- In the last 14 years, a significant reduction in ICU mortality due to severe community-acquired pneumonia was observed.
- In severe non-pneumococcal community-acquired pneumonia, early antibiotic administration and combination therapy were associated with a significant improved survival.
- A lower mortality was observed following early administration of combination therapy either in the subgroups of patients with shock, under mechanical ventilation and without shock or requirement of mechanical ventilation.
- The most frequent etiologies in severe CAP in the present cohort were *Staphylococcus aureus* followed by *Legionella pneumophila* and *Haemophilus influenzae*.

## Abbreviations

APACHE: Acute Physiology and Chronic Health Evaluation
CAP: Community-acquired pneumonia
CI: Confidence interval
ICU: Intensive care unit
IDSA/ATS: Infectious Diseases Society of America/American Thoracic Society
IMV: Invasive mechanical ventilation
OR: Odds ratio
SAPS: Simplified Acute Physiology Score
SCAP: Severe community-acquired pneumonia

## References

[1] Mandell LA, Wunderink RG, Anzueto A, Bartlett JG, Campbell GD, Dean NC (2007). Infectious Diseases Society of America/American Thoracic Society consensus guidelines on the management of community-acquired pneumonia in adults. Clin Infect Dis..

[2] Dombrovskiy VY, Martin AA, Sunderram J, Paz HL (2007). Rapid increase in hospitalization and mortality rates for severe sepsis in the United States: a trend analysis from 1993 to 2003. Crit Care Med..

[3] Waterer GW, Kessler LA, Wunderink RG (2006). Delayed administration of antibiotics and atypical presentation in community-acquired pneumonia. Chest..

[4] Bordon J, Aliberti S, Duvvuri P, Wiemken T, Peyrani P, Natividad I (2013). Early administration of the first antimicrobials should be considered a marker of optimal care of patients with community-acquired pneumonia rather than a predictor of outcomes. Int J Infect Dis..

[5] Gaieski DF, Edwards JM, Kallan MJ, Carr BG (2013). Benchmarking the incidence and mortality of severe sepsis in the United States. Crit Care Med..

[6] Kumar G, Kumar N, Taneja A, Kaleekal T, Tarima S, McGinley E (2011). Nationwide trends of severe sepsis in the 21st century (2000–2007). Chest..

[7] Vallés J, Palomar M, Alvárez-Lerma F, Rello J, Blanco A, Garnacho-Montero J (2013). Evolution over a 15-year period of clinical characteristics and outcomes of critically ill patients with community-acquired bacteraemia. Crit Care Med..

[8] The Acute Respiratory Distress Syndrome Network (2000). Ventilation with lower tidal volumes as compared with traditional tidal volumes for acute lung injury and the acute respiratory distress syndrome. N Engl J Med.

[9] Gattarello S, Borgatta B, Solé-Violán J, Vallés J, Vidaur L, Zaragoza R (2014). Decrease in mortality in severe community-acquired pneumococcal pneumonia: impact of improving antibiotic strategies (2000–2013). Chest..

[10] Bodí M, Rodríguez A, Solé-Violán J, Gilavert MC, Garnacho J, Blanquer J (2005). Antibiotic prescription for community-acquired pneumonia in the intensive care unit: impact of adherence to Infectious Disease Society of America guidelines on survival. Clin Infect Dis..

[11] Knaus WA, Draper EA, Wagner DP, Zimmerman JE (1985). APACHE II: a severity of disease classification system. Crit Care Med..

[12] Moreno RP, Metnitz PG, Almeida E, Jordan B, Bauer P, Campos RA (2005). SAPS 3: from evaluation of the patient to evaluation of the intensive care unit. Part 2: Development of a prognostic model for hospital mortality at ICU admission. Int Care Med.

[13] Langmore SE, Skarupski KA, Park PS, Fries BE (2002). Predictors of aspiration pneumonia in nursing home residents. Dysphagia..

[14] Lim WS, van der Eerden MM, Laing R, Boersma WG, Karalus N, Town GI (2003). Defining community acquired pneumonia severity on presentation to hospital: an international derivation and validation study. Thorax..

[15] Yoshimoto A, Nakamura H, Fujimura M, Nakao S (2005). Severe community-acquired pneumonia in an intensive care unit: risk factors for mortality. Intern Med..

[16] Arnold FW, Brock GN, Peyrani P, Rodríguez EL, Díaz AA, Rossi P (2010). Predictive accuracy of the pneumonia severity index vs CRB-65 for time to clinical stability: results from the Community-Acquired Pneumonia Organization (CAPO) International Cohort Study. Respir Med..

[17] Walden AP, Clarke GM, McKechnie S, Hutton P, Gordon AC, Rello J (2014). Patients with community-acquired pneumonia admitted to European intensive care units: an epidemiological survey of the GenOSept cohort. Crit Care..

[18] Baddour LM, Yu VL, Klugman KP, Feldman C, Ortqvist A, Rello J (2004). Combination antibiotic therapy lowers mortality among severely ill patients with Pneumococcal bacteraemia. Am J Respir Crit Care Med..

[19] Rello J, Gattarello S, Souto J, Sole-Violan J, Valles J, Peredo R (2013). Community-acquired Legionella pneumonia in the intensive care unit: impact on survival of combined antibiotic therapy. Med Intensiva..

[20] Dulhunty JM, Webb SA, Paterson DL, Bellomo R, Myburgh J, Roberts JA (2010). A survey of antibiotic prescribing practices in Australian and New Zealand intensive care units. Crit Care Resusc..

[21] Sligl WI, Asadi L, Eurich DT, Tjosvold L, Marrie TJ, Majumdar SR (2014). Macrolides and mortality in critically ill patients with community-acquired pneumonia: a systematic review and meta-analysis. Crit Care Med..

[22] Torres A, Garau J, Arvis P, Carlet J, Choudhri S, Kureishi A (2008). Moxifloxacin monotherapy is effective in hospitalized patients with community-acquired pneumonia: the MOTIV study—a randomized clinical trial. Clin Infect Dis..

[23] Heffelfinger JD, Dowell SF, Jorgensen JH, Klugman KP, Mabry LR, Musher DM (2000). Management of community-acquired pneumonia in the era of pneumococcal resistance: a report from the Drug-Resistant Streptococcus pneumoniae Therapeutic Working Group. Arch Intern Med..

[24] Grossman RF, Hsueh PR, Gillespie SH, Blasi F (2014). Community-acquired pneumonia and tuberculosis: differential diagnosis and the use of fluoroquinolones. Int J Infect Dis..

[25] Kumar A, Roberts D, Wood KE, Light B, Parrillo JE, Sharma S (2006). Duration of hypotension before initiation of effective antimicrobial therapy is the critical determinant of survival in human septic shock. Crit Care Med..

[26] Nobre V, Sarasin FP, Pugin J (2007). Prompt antibiotic administration and goal-directed hemodynamic support in patients with severe sepsis and septic shock. Curr Opin Crit Care..

[27] Rautanen A, Mills TC, Gordon AC, Hutton P, Steffens M, Nuamah R (2015). Genome-wide association study of survival from sepsis due to pneumonia: an observational cohort study. Lancet Respir Med..

[28] Waterer GW (2014). Better outcomes from pneumococcal pneumonia: how good is your care?. Chest..

